# The value of Cone-Beam CT-guided radiofrequency ablation in the treatment of pulmonary malignancies (≤3 cm)

**DOI:** 10.1042/BSR20181230

**Published:** 2019-02-26

**Authors:** Zi-jun Xiang, Yi Wang, En-fu Du, Lin Xu, Bin Jiang, Huili Li, Yun Wang, Ning Cui

**Affiliations:** 1 Center of Liver-Biliary-Pancreatic, Taihe Hospital, Shiyan City, Hubei Province; 2 Department of Radiology, Yunxi People’s Hospital, Shiyan City, Hubei Province; 3 Medical Imaging Center-Intervention Room, Taihe Hospital, Shiyan City, Hubei Province; 4 Center of Liver-Biliary-Pancreatic, Taihe Hospital, Shiyan City, Hubei Province

**Keywords:** Cone-beam CT, pulmonary malignancies, radiofrequency ablation

## Abstract

The aim of this study is to explore the safety and efficacy of Cone-Beam computed tomography (CBCT) guided radiofrequency ablation (RFA) in the treatment of pulmonary malignancies. Thirty-one patients with pulmonary malignant tumors (≤3 cm in diameter) were enrolled to this study. Total 43 CBCT guided RFA treatments were performed, including 7 patients undergoing multiple treatments. The target tumor puncture success rate, tumor remission rate, postoperative cumulative survival rate, tumor-free survival rate and complication rate were analyzed. All 43 CBCT guided RFA procedures successfully punctured the target tumors. Complications included five cases of pneumothorax and three cases of hemoptysis. For the 31 patients who underwent CBCT guided RFA, the 1- and 2-year cumulative survival rates were 80.6 and 54.8%, respectively. The 1- and 2-year disease-free survival rates were 54.8 and 32.3%, respectively. The 1-, 3- and 6-month remission rates were 78.4, 68.7 and 63.3%, respectively. The average cumulative radiation dose and average effective radiation dose were 194.62 ± 105.51 mGy and 5.41 ± 3.45 mSv, respectively. CBCT help to shorten the operation time, reduce the unnecessary interventions and also reduce the incidence of complications. CBCT guided RFA is one safe and efficacious treatment for pulmonary malignancies.

## Introduction

Cancer is the leading cause of death worldwide, accounting for 8.8 million deaths in 2015. Lung cancer is the leading cause of cancer mortality for men worldwide and for women in developed countries. In developing countries, the mortality rate due to lung cancer is only less than breast cancer [1,2]. For the treatment of lung cancer, surgery, chemotherapy and radiotherapy are still the primary choice in the clinic. In recent years, immunotherapy and targeted therapy are gradually developed and bring great potential for these patients, such as cytokine-induced killer cells [3,4].

Radiofrequency ablation (RFA) is one new developed and minimally invasive cancer therapy technique. Studies showed that it was widely used to treat various tumors, such as liver tumors, renal tumors [5,6]. The efficacy and safety of RFA for lung cancers are also evaluated and studies suggested that RFA was one safe and efficient treatment for the patients with lung cancers [7]. It is especially available for patients, who do not have the opportunity to undergo surgery, high-risk patients unsuitable for surgery and for patients with pulmonary metastasis and refused to receive surgery [8].

Computed tomography (CT) is the most widely used image guidance technique in clinical situations. Cone-Beam CT (CBCT) is an image guidance technique that has grown quickly in recent years. The CBCT system provides projection radiography, fluoroscopy, digital subtraction angiography (DSA) and volumetric CT capabilities in a single patient setup within the interventional suite. It can obtain both real-time fluoroscopy capability as well as CT images with 3D (three-dimensional) reconstruction. These advantages make the performance of intra-procedural volumetric imaging convenient, which does not need the patient transportation. Few studies indicated that CBCT was a very effective technique for lung biopsies in clinical settings [9]. In this study, we will assess the safety and efficacy of using CBCT guided RFA to treat pulmonary malignancies of diameter less than or equal to 3 cm.

## Materials and methods

### Data collection

This study was approved by The Ethics Committee of Taihe Hospital. Participants have provided their written informed consent to participate in this study. The research has been carried out in accordance with the World Medical Association Declaration of Helsinki.

Clinical data were collected from Radiology Department of Taihe Hopsital of Shiyan, Hubei Province between November 2009 and March 2016. The patients who underwent CBCT guided RFA treatment of pulmonary malignant tumors with diameters less than or equal to 3 cm were enrolled. A total of 31 patients (16 males and 15 females) were included in the study. The age of the patients ranged from 28 to 80 years and the average age was 52.6 years. A total of 43 CBCT guided RFA treatments were performed, including 7 patients undergoing multiple treatments. A total of 37 tumors were targeted in the study. The diameters of these tumors ranged from 1 to 3 cm and the mean size was 1.93 cm.

All patients underwent biopsies to confirm pathological diagnoses prior to the CBCT guided RFA treatment. The biopsies indicated that 24 patients had primary lung cancer, including 20 cases of non-small cell lung cancer and 4 cases of small cell lung cancer. In seven patients, the lung cancer was due to metastases of other cancers and the primary cancer had been controlled.

Biopsy methods included percutaneous transthoracic biopsy in 29 patients and bronchoscopy biopsy in 2 patients. All patients in the study were unable to tolerate surgery. Any patients with severe bleeding tendencies (platelet counts below 50 × 10^9^ per L) and severe clotting disorders (prothrombin time greater than 18 s, prothrombin activity less than 40%) were excluded from the study.

### Equipment

The CBCT guidance was achieved using either the GE INNOVA 3100 IQ (Fairfield, CA, USA) or the PHILIPS 1250-FD20 (Amsterdam, Netherlands). The CBCT scan covered a spatial range of 225 cm x 225 cm x 185 cm. The scan time used was either 5,10 or 20 s and depended on the number of images collected. A single 240° rotation of the C-arm system collected 310 images (at 30 frames/s) or 620 images (at 60 frames/s). The volume data obtained by the rotary scan was transferred to the post-processing station (XperGuide). The image reconstruction took 5 s and provided an isotropic 3D volume image. The RFA was performed using the Model 1500X RF Generator and Starburst XL ablation needles, both from RITA Medical Systems (Mountain View, CA, USA).

### CBCT guided RFA treatment procedure

The general condition of all patients was assessed prior the operation. This assessment included history of infection, cardiopulmonary insufficiency, hemorrhage disorder and the use of anticoagulants and bronchodilators. All patients underwent preoperative fasting including no solid food for 8 h and no water for 4 h. Pethidine (50 mg) was injected intramuscularly 30 min before surgery.

The size and location of the tumors and the structures adjacent to the tumors were assessed according to the recent chest CT scans of the patients. The operating position for each patient (supine, prone or lateral) was chosen according to the location of the tumor in the lungs to minimize the distance between the skin puncture site and the lung lesion and avoid the ribs, large blood vessels, pulmonary bullae, tracheas, lung fissures and scapulae.

The arms of patients were positioned above the head and a metal marker with grids was placed at the skin puncture site to help locate the lung lesion. The patients were asked to perform breathing exercises 1 day before surgery to help establish the depth of each breath (tidal volume). Breathing exercises included holding the breath for 10 to 15 s, 30 times a day.

During the operations, rotational scanning was conducted using CBCT. The image data obtained from the scan was transmitted to an AW workstation (General Electric Company) for 3D reconstruction. The reconstruction of the multi-planar CT images clearly showed the location, shape and size of the tumor. Using the workstation’s measuring tool, an insertion path was drawn from the body surface to the target lesion in the lung. The depth was measured and an appropriate puncture site on the surface of body was chosen on the metal marker grid. The puncture point on the patient’s body surface was marked and then sterilized. Lignocaine (2% w/v) was applied for local anesthesia via a needle from the puncture point to the pleural wall. The position of the needle was then used to measure the distance between the tip of the needle and the target tumor.

The ablation needle was inserted via the marked puncture point. The patient was told to hold his or her breath when the needle reached the muscular layer. The ablation needle was then quickly advanced to the distance previously measured and then fixed. The position of the ablation needle (in both the closed and the open position) relative to the tumor was checked using CBCT and adjusted as necessary. RFA was performed when it was confirmed that the open ablation needle was positioned appropriately relative to the tumor.

If the patient was in significant pain during the procedure, 50 mg of pethidine was administered intramuscularly. After the RFA operation, CBCT was used to evaluate the result of the ablation of the tumor and to identify any complications, such as hemorrhage and pneumothorax.

### Ablation parameters

The power of the radiofrequency generator was 90 watts. The initial ablation temperature was 70°C, which gradually increased to 90°C as the ablation started. In order to ensure satisfactory results of the ablation, the electrode was extended 0.5 cm beyond the edge of the tumor. The ablation time was shown in Table 1

**Table 1:** Effective ablation time

Tumor size	Effective ablation time (Target temperature ≥70°C) min		
	2 cm	3 cm	4 cm
≤1 cm	10	–	–
>1 cm, ≤2 cm	–	15	–
>2 cm, ≤3 cm	–	5	15

The dose area product (DAP) (cGy·cm^2^) applied to each patient in each operation was recorded. The effective dose (mSv) was calculated by multiplying the DAP by a conversion coefficient (*k* = 0.14 mSv/Gy cm^2^)according to the Monte-Carlo transform coefficient model [10].

### Evaluation of the response of the pulmonary malignancies to the CBCT guided RFA treatment

The response of the tumors to the CBCT guided RFA treatment was evaluated using a modified Response Evaluation Criteria for Solid Tumors (RECIST) [11]. CBCT scans were conducted 1, 3, 6 months and then every 6 months after surgery to monitor the tumors. The response categories included complete remission, partial remission, disease stability and disease progression (Table 2).

**Table 2:** Modified RECIST used to evaluate tumor response to treatment

Response category	CT mass size (RECIST)	CT mass quality
Complete remission (any 2)	Lesion disappearance or scar <25% original size	Cyst or cavity formation
Partial remission (any 1)	Decrease of >30% in LD^A^ of target lesion^B^	Low density of entire lesion; Central necrosis or central cavitation with liquid density
Stable lesion (any 1)	Decrease of <30% in LD of target lesion	Mass solid appearance, no central necrosis or cavity
Progression (any 2)	Increase of >20% in LD of target lesion	Solid mass, invasion adjacent structures

A: Target lesions represent tumors treated with CBCT RFA

B: LD is the largest diameter of target lesions.

### Statistical analysis

The survival rate was calculated by the Kaplan-Meier probability curve. The SPSS version 16.0 software (SPSS, Chicago, IL USA) was used to perform a statistical analysis of the postoperative cumulative survival rate, remission rate and operation complication rate.

## Results

### The efficacy of CBCT guided RFA in the treatment of pulmonary malignancies

During the operation, the median puncture time was 11 min (5–28 min). The median number of puncture adjustments was 4 (3–6 times). Puncture accuracy was 3 ± 0.3 mm (0–9 mm). Median ablation time was 34.5 min (33.7 ± 7.5 min). The average cumulative radiation dose and average effective radiation dose were 194.62 ± 105.51 mGy and 5.41 ± 3.45 mSv, respectively.

**Figure 1:**
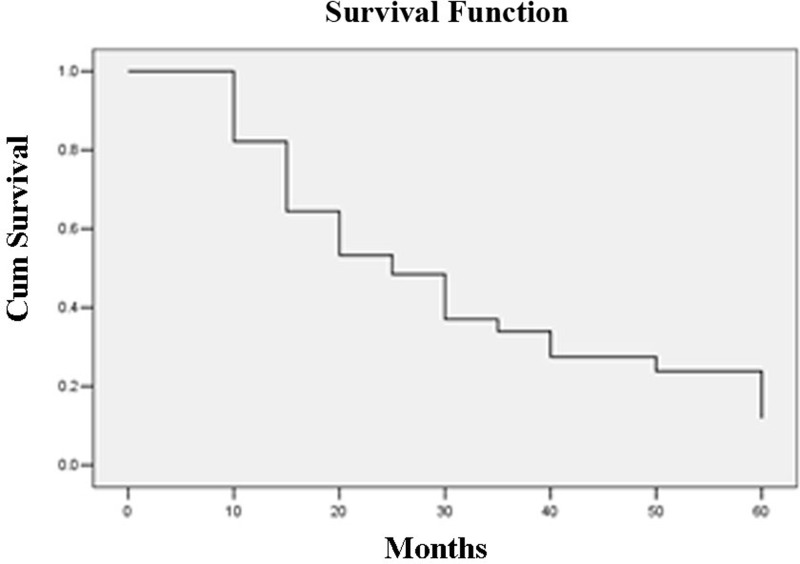
Cumulative survival rate for patients with pulmonary malignancies after Cone-Beam CT-guided RFA

The tumor response to the CBCT guided RFA treatment were conducted between 3 and 82 months post-treatment. Of the 31 patients in this study, 22 died and 9 survived. According to the life table method, the calculated 1- and 2-year survival rates were 80.6 and 54.8%, respectively. The 1- and 2-year local control rates (CR + PR) were 78.4% (29/37) and 62.5% (20/32), respectively. The median survival time was 25 months (95% CI, 28.1–68). The 1- and 2-year survival rates for primary lung cancer were 79.2% (19/24) and 45.8% (11/24), respectively (Figure 1).

During the operation, some complication happened. There were five patients with pneumothorax. The incidence rate was 11.6%(5/43). All patients were in spontaneous remission and recovered postoperatively. There were three patients with bleeding. The incidence rate was 6.9%(3/43). Bleeding is caused by incomplete needle burn after RFA and is generally self-healing.

## Discussion

CT is the most commonly used radiological guidance method in lung biopsy or pulmonary ablation. CBCT is a new developed interventional radiology technology, which includes the C-arm rotary motion, flat panel detector acquisition and CT reconstruction technologies. The technique involves the use of DSA to capture the images, followed by transfer of image data to a workstation for volume reconstruction, multiple planar reconstruction and maximum intensity projection reconstruction, which provides 3D CT-like stratified images. Cazzato et al. reported that compared with CT guidance, CBCT allowed faster lung RFA irrespective of the size of the lung lesion. CBCT guided RFA also resulted in a lower rate of pneumothoraxes than with CT-guided RFA (37.5% for CBCT vs 42.8% for CT). The recurrence of tumors was also lower in patients who underwent CBCT guided lung RFA (11.7 vs 15.8%) [12]. These data suggest that CBCT had advantages in RFA than CT.

In this study, we achieved a puncture success rate of 100% in the 43 RFA treatments for 31 patients through CBCT, which is consistent with literatures. Jin et al. did a retrospective analysis of 71 cases of CBCT-guided biopsies of pulmonary nodules of diameter ≤3cm) and found that the puncture success rate was also 100% and a diagnostic accuracy was 98.4% [13]. Choo’s study also showed a 100% puncture success rate and 98% diagnostic accuracy [9]. So, literatures and our study all supported that CBCT was an ideal and feasible imaging guidance method for lung RFA

The C-arm design of the CBCT equipment provides greater convenience than conventional CT for the positioning of patients and surgeons during operations. At present, it is difficult to measure the radiation dosage of CBCT due to its complexity. Unlike the traditional multi-slice spiral CT, there are no general accepted dose measurement standards for CBCT. Koyama et al. reported that a single effective radiation dose of CBCT chest scan is 0.92 mSv, whereas the effective radiation dose of a conventional chest CT scan is 2.71 ± 1.14 mSv [14,15]. From the perspective of a single chest scan, CBCT’s radiation dose is less than the conventional CT. Hwang et al. reported that the average effective radiation dose for CBCT guided lung biopsy is 4.6 mSv [16]. Jin et al. also stated that the cumulative radiation dose is 271 ± 116 mGy using CBCT-guided lung biopsy [7]. In this study, the average cumulative dose and average effective dose were 194.62 ± 105.51 mGy and 5.41 ± 3.45 mSv, respectively. The cumulative radiation dose was lower that Jin’s study, however, the average effective radiation dose was similar to Hwang’s study. So, we found that the radiation dosage used in CBCT guidance was lower as compared with conventional CT guidance, which is consistent with our previous studies [17,18].

The disadvantage of CBCT is a lack of spatial resolution. There is still a significant gap compared with conventional CT [19]. It is difficult for CBCT to clearly distinguish the anatomical relationship of the tumor mass from the heart and large vessels, which may lead to huge risks during surgery. Additional conventional CT images are needed as a guide for such operations. However, lung tissue has natural good contrast due to rich gas, which makes CBCT clearly distinguish the anatomical relationship between lung mass and surrounding tissues. Higashihara reported that, there was no significant difference between CBCT and conventional CT in the detection rate of pulmonary nodules, which are equal or greater than 8 mm in diameters [20]. Therefore, we believe that CBCT working as image guidance for lung tumor operation is acceptable even though it has insufficient spatial resolution.

Zhou et al. reported that the median size of pulmonary malignancies treated with RFA was 2.2 cm (1.7–5.2 cm). CT-guided operations had an average survival time of 23 months (8.6–33 months). The 1-year survival rate ranged from 63 to 85% and the 2-year survival rate ranged from 55 to 65% [21]. Our data showed that the median size of pulmonary malignancies treated with RFA was 1.93 cm (1.0–3.0 cm). The median survival time was 25 months (95% CI, 10.1–39.9 months). The median tumor free period was 17 months (95% CI, 5.6–28.4 months). The 1- and 2- year survival rates were 80.6 and 54.8%, respectively. Simon et al. evaluated long-term survival rate and local tumor progression-free rate for patients who underwent CT-guided RFA for primary or metastatic lung cancer. They found that for patients who had stage I non small-cell lung carcinoma (NSCLC) (tumor diameter ≤3 cm), the 1- and 2-year survival rates were 78 and 57%, respectively and the 1- and 2-years local tumor progression-free rates were 83 and 64%, respectively [8]. In this study, the 2-year survival rate of primary lung cancer was 48.5%, which was lower than the 57% observed by Simon. This could be due to the primary lung cancer cases. In our study, the patients cover NSCLC and small-cell lung carcinoma (SCLC), which have different prognosis. There was no significant difference in local tumor progression-free rate. So, although the image guidance methods are different, there is no difference in prognosis in lung tumors with diameter ≥1cm using CBCT guided RFA or conventional CT-guided RFA.

RFA is a relatively safe minimally invasive treatment method for pulmonary tumors. In this study, seven patients required multiple RFA treatments due to tumor progression All patients tolerated multiple RFA. There is no severe complications, such as pneumothorax. Studies showed that pneumothorax was the most common complication in patients who underwent lung tumor RFA treatment. The incidence rate is between 10 and 60% and the majority of pneumothorax is self-confinement and did not require medical management [21,22]. Not more than 10% of patients needed thoracentesis aspiration or closed chest drainage. The incidence of intra-pulmonary hemorrhage was approximately 3–8% [21,23]. During the process of surgical puncture, lung injury may occur and lead to intra-pulmonary hemorrhage. However, the process of lung ablation can stop the hemorrhage. In this study, complications included three cases of hemoptysis (6.9%) and five cases of pneumothorax (11.6%). There was no delayed onset of pneumothorax (72 h post operation). None of the complications needed further medical management. There was no significant difference in CBCT-guided RFA in terms of complications compared with CT-guided RFA.

In addition, we believed that, CBCT had several unique advantages. First, CT-like images can be directly obtained to clearly display the location of tumor and ablation needle. Puncture accuracy is 3 ± 0.3 mm(0–9 mm). Compared with conventional CT, secondary reconstruction time is not needed., The median puncture time was 12 min. The median number of puncture adjustments was 4 times (3–6 times), which not only prolonged the operation time, but also increased the radiation dose. CBCT and radiological intervention were operated in the operating room, which provide more life support for the patients under emergency. The C arm design of CBCT can provide greater convenience for the operation and rescue.

## Conclusions

In conclusion, CBCT is the product of the combination of multisystem and multi-information platform. CBCT guided lung malignancies RFA treatment is a highly safe and effective technique compared with the most commonly used CT-guided RFA. The 3D real-time reconstruction of the CBCT allows tissue lesions to be observed at any angle, thus providing detailed and useful imaging information. The 3D images from CBCT make it clear to identify the lung lesions and their surrounding tissues. The images also help the doctor to set puncture paths and measure the puncture depth and angle. Through providing the real time 3D images during operations, CBCT help to shorten the operation time, reduce the unnecessary interventions and also reduce the incidence of complications.

## Abbreviations

3D: three-dimensional
CBCT: Cone-Beam computed tomography
CT: computed tomography
DAP: dose area product
DSA: digital subtraction angiography
NSCLC: non small-cell lung carcinoma
RECIST: response evaluation criteria for solid tumors
RFA: radiofrequency ablation
SCLC: small-cell lung carcinoma
